# Interplay of Total Antioxidant Capacity and Oxidative Stress Hydroperoxides with Circulating Biomarkers of Inflammation and Iron Status According to Oral Contraception Use

**DOI:** 10.3390/antiox15040523

**Published:** 2026-04-21

**Authors:** Sabina Cauci, Cinzia Buligan, Patrizia Nacci, Lorenza Driul, Francesco Curcio, Gianluca Tell, Maria Pia Francescato

**Affiliations:** 1 Department of Medicine, School of Medicine, University of Udine, Piazzale Kolbe 4, 33100 Udine, Italy; patrizia.nacci@uniud.it (P.N.); lorenza.driul@uniud.it (L.D.); francesco.curcio@uniud.it (F.C.); gianluca.tell@uniud.it (G.T.); mariapia.francescato@gmail.com (M.P.F.); 2 Institute of Dermatology, Azienda Sanitaria Universitaria Friuli Centrale (ASUFC), 33100 Udine, Italy; cinzia.buligan@asufc.sanita.fvg.it; 3 Clinic of Obstetrics and Gynecology, Santa Maria Della Misericordia, Friuli Centrale Healthcare University Hospital (ASUFC), 33100 Udine, Italy; 4 Department of Laboratory Medicine, Institute of Pathology, Santa Maria Della Misericordia, Friuli Centrale Healthcare University Hospital (ASUFC), 33100 Udine, Italy

**Keywords:** oxidative stress, antioxidant defence capacity, hydroperoxides, lipid peroxidation, iron status, serum iron, serum ferritin, transferrin, transferrin saturation, soluble transferrin receptor, sTfR-F index, females, young women, contraception, smoke, coffee, tea, BMI

## Abstract

We evaluated the interplay between systemic total antioxidant capacity (TAC), oxidative stress (OS) (lipid hydroperoxides), inflammation, iron status, and oral contraception (OC) use in 182 healthy 23-year-old women (76 OC-users, and 106 non-OC-users). In all women, blood TAC (FORD units) values were significantly inversely associated with OS (FORT units), high-sensitivity C-reactive protein (hsCRP), and transferrin; and positively associated with transferrin saturation (TfS%). No significant associations were observed for hemoglobin, hematocrit, red blood cells, serum iron, soluble transferrin receptor (sTfR), sTfR/log(ferritin) ratio (sTfR-F index), ferritin, folate, uric acid, or creatinine. OS hydroperoxides were positively associated with hsCRP and transferrin, and inversely associated with TfS%. sTfR was positively correlated with hydroperoxides in non-OC-users and with folate in all women and non-OC-users, but was not associated with hsCRP in any group. The combined abnormal condition of low TAC and elevated OS (*n* = 71) was significantly more frequent among OC-users (OR = 39.0), women with hsCRP ≥ 3 mg L^−1^ (OR = 10.1), transferrin ≥ 330 mg dL^−1^ (OR = 6.58), and smokers (OR = 3.76). OC use modulated the TAC/OS balance and inflammation. Low TAC and elevated OS may impact health status. Enhanced TAC/OS knowledge may increase awareness of effects of OC use among fertile-age women. Ferritin was independent of TAC/OS status and OC use, supporting its reliability as an iron biomarker.

## 1. Introduction

Oxidative stress (OS) is a complex and vital component of the human body’s response to various external and physiological stimuli [1,2]. The cellular and molecular events regulating the interactions between the various players in the oxidative stress process are the subject of several ongoing investigations focusing on women, because they can impact female health [3,4,5,6,7,8,9], and may be modulated, at least partially, by lifestyle and dietary habits including antioxidant supplementation [2,9,10,11], and exposure to environmental pollutants [12,13].

Oxidative stress arises from an imbalance between the overproduction of free radicals, especially reactive oxygen species (ROS), including mainly superoxide anion (O_2_^•-^), hydroxyl radical (HO^•^), and hydrogen peroxide (H_2_O_2_), and the body detoxification systems. These defence mechanisms consist of enzymatic and non-enzymatic factors such as vitamins A, C, D, E, carotenoids, flavonoids, eumelanin (a brown-black skin pigment that confers photoprotection from solar UV radiation), reduced glutathione (GSH), glutathione peroxidase (GPx), catalase (CAT), superoxide dismutase (SOD), and heme “oxygenase 1” (HO-1), which collectively constitute the antioxidant defence capacity [1,2,10].

When an excess of free radical occurs, they can attack various molecules, generating hydroperoxides (ROOH), particularly lipid peroxides, which can be measured in the blood [14,15,16], and can cause damage to proteins and DNA [1,2,17]. Oxidative stress is involved in the pathogenesis of several diseases, including cardiovascular, inflammatory, and neurodegenerative diseases, and tumours [1,2,3,4,5,6,7,8,9,18]. However, free radicals also have physiological and beneficial roles, contributing to the regulation of cell functions and inflammatory responses [1,2].

In the context of reproductive-aged women, studies have demonstrated that the use of oral contraception (OC) and other external hormonal treatments are associated with significantly increased oxidative stress [14,15,16,17,19,20]. Furthermore, increasing evidence suggests that OC use promotes chronic low-grade inflammation, typically assessed through increased levels of high-sensitivity C-reactive protein (hsCRP) [21,22,23]. A recent study showed that, in healthy women, increased hydroperoxides and hsCRP levels are closely interrelated [16]. However, the underlying mechanisms, as well as the role of the antioxidant defences, their relationships with other systemic biomarkers, and the potential implications, remain unclear.

A critical factor in this balance is body iron, which is implicated in oxidative stress primarily through the Fenton reaction. In this process, Fe^2+^ catalyses the transformation of H_2_O_2_ in the aggressive hydroxyl radical, that causes deleterious oxidative damage to DNA, proteins, and membrane lipids, causing lipid peroxidation [2,24,25,26]. Despite this, the multifaceted interplay between systemic antioxidant capacity, oxidative stress, and iron status indicators in women remains poorly understood.

Iron deficiency (ID) is one of the most common nutritional deficiencies worldwide, particularly affecting women of reproductive age [27,28,29,30,31]. This condition adversely affects overall well-being and health status, causing reduced quality of life [27,28,29,30,31]. Prolonged or severe ID may result in iron deficiency anaemia (IDA), a pathological condition associated with multiple clinical consequences, including increased risk of morbidity and need for transfusion in surgical patients [29,30,31,32,33,34,35].

In reproductive-aged women, ID is much more frequent than in men [27,28]; this is primarily attributed to high menstrual blood loss and/or blood donation, particularly in those women with inadequate dietary iron intake and low body mass index [29,30,31,34,35]. Consequently, the early recognition and prompt treatment of ID are crucial for maintaining optimal health in fertile women [29,30,31,32,33,34,35].

Evaluation of body iron status and guidance for iron supplementation can be achieved through several blood biochemical markers [27,28,29,30,31,34,36]. Iron deficiency is typically characterised by low serum iron, low percentage of transferrin saturation (TfS%), and low ferritin, and conversely by elevated transferrin, soluble transferrin receptor (sTfR), and sTfR/log(ferritin) ratio (sTfR-F index) [27,28,33,34,35,36,37,38]. However, the reliability of these markers can be compromised by circadian variations (especially for serum iron), physiological changes, and/or numerous clinical or subclinical disorders, especially inflammation, that may modulate the levels of several ID blood biomarkers [36,37,38,39,40].

Currently, the most widely used indicators of iron deficiency are low serum ferritin (reflecting body iron stores) [27,28,32,33,36,37,38,39,40,41], low percentage of transferrin saturation (TfS%, a marker of iron availability) [27,28,30,36], and increased soluble transferrin receptors (sTfRs) a biomarker linked to elevation of the receptor present on erythroid precursors in the case of iron deficiency [36,37,38,42,43]. Soluble TfR is also a sensitive marker of stimulated erythropoiesis [36,43] and, importantly, has the advantage, compared to transferrin by itself, of not being an acute phase protein, making it less influenced by inflammation [36,42,43]. Moreover, sTfR has low biological variability and remains stable in several conditions such as after physical exercise [38,42,43]. In recent years, the sTfR-F index, which combines sTfR and log ferritin concentrations, has been widely used as a measure of iron deficit, being more sensitive and specific than sTfR or ferritin alone [36,42,43].

Iron homeostasis is tightly regulated at the cellular and systemic levels [39,40,41]. Body iron is required not only for erythropoiesis, but it is also linked to the immune/inflammatory response and oxidative metabolism through multiple mechanisms [27,28,30,36,39,40,41]. While the relationships of iron homeostasis and inflammation mechanisms have been rather widely studied, much less is known about its complex relationships with systemic antioxidant defence and oxidative stress [30,44], particularly in young adult women according to OC use.

Therefore, the aim of this study was to evaluate the interplay of systemic antioxidant defence capacity, oxidative stress, inflammation, and iron status biomarkers in a population of young healthy menstruating women, particularly in the context of OC use. We also aimed to identify the characteristics of women with *versus* without an abnormal TAC/oxidative stress status, as defined by the combination of low TAC and elevated OS (hydroperoxides) values. We further assessed which blood iron status biomarkers were independent of TAC/OS status and OC use, thus being reliable to use for diagnosis of iron homeostasis in women of reproductive age.

## 2. Materials and Methods

### 2.1. Design and Population

The study was conducted in accordance with the ethical principles of the Declaration of Helsinki, and was approved by the Local Institutional Ethics Committee, Department of Medicine, University of Udine, authorisation number 554.

Non-pregnant and non-lactating Italian women aged 18 to 40 years were recruited as volunteers in Udine, mainly among students and employees of the University of Udine, all self-reporting good health status. None of the women reported adherence to vegetarian or vegan diets. None were using semaglutide or analogous hypoglycaemic agents. Furthermore, chronic and/or acute pathological conditions such as thalassemia and other hematologic disorders, coeliac disease, diabetes mellitus, immunologic disorders, cardiovascular diseases, tumours, current infections, present or past amenorrhea or hypermenorrhoea, and other reproductive tract pathological conditions [16], were excluded based on a structured interview of voluntary women at entry into the study, and by review of a written standardised questionnaire collecting demographic, lifestyle, and medical history information filled out by each participant before blood sample collections [16]. Information regarding coffee and smoking habits, as well as nutritional supplement use was retrieved from a two-week-long daily questionnaire.

For inclusion in the non-OC-users group, women had to have discontinued any hormonal treatment for more than 3 months or to have never used hormonal contraception [16]. For inclusion in the OC-users group women had to be using a combined oral contraceptive pill for at least 3 months [16]. All combined contraceptive pills were monophasic and contained different types of progestin as follows: 45 pills contained gestodene, 12 desogestrel, 10 drospirenone, 5 levonorgestrel, and 4 cyproterone. Thus, among the 76 OC-users enrolled in the study, 57 (75%) women were using third generation pills (containing gestodene and desogestrel), 14 (18%) fourth generation pills (drospirenone and cyproterone), and 5 (7%) second generation pills (levonorgestrel). No woman had ever used an intrauterine device (IUD).

Regarding the use of nutritional supplements, statistical analysis was performed after categorisation (yes/no) [15], since participants consumed a wide variety of different commercial products, varying in regard to composition, quantity, and consistency of assumption (occasional, intermittent, or continuous).

Participants were requested to avoid alcohol and supplement consumption, as well as vigorous physical activity or heavy working, for 24 h before blood collection [15,16]. The use of anti-inflammatory drugs or antibiotics within the previous 14 days [16] was considered an exclusion criterion.

### 2.2. Measurements

Finger capillary and arm venous blood samples were collected after overnight fasting from seated subjects in the morning, avoiding the menstrual bleeding days, as previously described [16,21,22,37,38]. Processing and analysis of venous blood samples were performed at the Department of Laboratory Medicine (ASUFC) according to appropriate standardised laboratory procedures. The following biomarkers were measured: haemoglobin (Hb), red blood cells (RBC), white blood cells (WBC), haematocrit (Hct), serum iron (reference interval 50–150 μg dL^−1^), ferritin, transferrin (reference interval 200–330 mg dL^−1^ for females), high sensitivity C-reactive protein (hsCRP), folate (reference interval 5.0–24 µg L^−1^), creatinine (reference interval 0.40–1.30 mg dL^−1^), and uric acid (reference interval 2.4–7.0 mg dL^−1^) [16,21,22,37,38].

No woman had Hb below 10 g L^−1^. Ferritin concentrations < 12 µg L^−1^ were considered as iron deficiency (ID) [37,38].

The percentage of transferrin saturation, TfS (%), was calculated according to the formula: TfS (%) = serum iron (mg L^−1^) × 70.9/Tf (g L^−1^) [36]. The hsCRP threshold of 3 mg L^−1^ was considered as a risky level for future cardiovascular diseases [16,21,45].

The sTfR concentrations (reference interval 0.83–1.76 mg L^−1^) were measured by using a Dade Behring (Brussels, Belgium) assay as described [37,38,42]. The ratio of sTfR to log ferritin (sTfR-F index) was subsequently calculated, and a cutoff of 1.5 was used to assess body iron depletion [36,37,38,42].

Total antioxidant capacity (TAC) was assessed using the Free Oxygen Radical Defence (FORD) assay (Callegari, Parma, Italy) in 50 μL capillary blood samples [14,19,46]. This assay evaluated the blood TAC resulting from various molecules such as ascorbic acid, total thiols (including glutathione), proteins like albumin and ceruloplasmin, bilirubin, polyphenols like flavonoids and tannins (but not uric acid) accounting for the majority of antioxidant activity [14,46]. The absorbance values of the samples were compared with a standard curve obtained using Trolox, a derivative of vitamin E commonly used as an antioxidant [46]. Results were expressed as FORD units, whereby 1 FORD unit corresponded to 1 mmol L^−1^ Trolox equivalent. Coefficients of intra- and inter-assay analytical variation were <5.0% [14,15,47]. Values < 1.07 FORD units were considered as low TAC according to manufacturer’s indications, and previous research [46].

Oxidative stress was assessed by measuring blood hydroperoxides, mainly consisting of lipid hydroperoxides [14,15,19,46] in 20 µL of capillary blood using the Free Oxygen Radical Test (FORT assay; Callegari, Parma, Italy), a rapid 6 min long colorimetric assay based on the ability of transition metals to catalyse the breakdown of hydroperoxides (ROOH) into radicals, according to the Fenton reaction [14,25,46]. Results were expressed as FORT units, whereby 1 FORT unit corresponded to 0.26 mg L^−1^ H_2_O_2_ [14,46]. Variations in the intra- and inter-assay were both <5.0%, roughly in accord with previous findings [14,47]. According to the Callegari manufacturer values < 300 FORT units are to be considered as optimal (normal) condition, values between 300 and 330 FORT units as latent oxidative stress (borderline), values ≥ 330 FORT units as overt strong oxidative stress level. Consequently, we used values ≥ 300 FORT units as indication of abnormally elevated oxidative stress levels. An elevated OS with simultaneously reduced TAC is considered a combination more firmly indicative of an altered oxidative status than each single biomarker by itself [46]; thus, we examined the abnormal oxidative condition consisting in low TAC < 1.07 FORD units combined with elevated OS hydroperoxides ≥ 300 FORT units according to cutoffs indicated by the manufacturer (Callegari, Parma, Italy).

Body mass index (BMI) was determined by weight (kg) divided by height squared (m^2^); BMI ≥ 23 kg m^−2^ was considered indicative of overweight for young Caucasian women according to the World Obesity Federation guidelines, which indicate BMI from 21 to 23 kg m^−2^ as optimal range values [48]. Overall, 6 study women had BMI ≥ 25 kg m^−2^, of which 4 were in the OC-users and 2 were in the non-OC-users group; no woman had BMI ≥ 30 kg m^−2^.

### 2.3. Statistical Analysis

For normally distributed variables, descriptive data were presented as mean and standard deviation (±SD), whereas skewed variables were expressed as median and interquartile range (25th to 75th percentile, IQR). The Mann–Whitney U test was used for comparison of continuous variables. For categorical variables, the differences in proportions between groups of women were assessed by odds ratios (ORs) and 95% confidence intervals (CIs), and statistical significance was assessed using χ^2^-test according to Pearson or Fisher test, as appropriate. Bivariate correlations were evaluated using Spearman’s rank correlation coefficient Rho (r_s_). All tests were two-sided. *p* values < 0.050 were considered statistically significant; *p* values between ≥0.050 and <0.100 were considered trends and indicated by ^, whereas *p* ≥ 0.100 were considered as totally non-significant (NS) data, that were not reported in correlation tables. Statistical analyses were performed using the Statistical Package for Social Sciences, version 28.0.1 (SPSS Inc., Chicago, IL, USA).

## 3. Results

The main demographic and anthropometric characteristics of the 182 study participants, as well as the comparison of 76 OC-users with 106 non-OC-users, are presented in Table 1. On average, women were 23.3 ± 4.3 years old, had a BMI of 20.8 ± 2.1 kg m^−2^, 79.1% had a university-level education, and almost all were nulliparous (97.8%). Overall, 22.7% of participants were smokers.

OC-users did not differ from non-OC-users in most characteristics, except for smoking, which was more frequent among OC-users (34.2% versus 14.4%, OR = 2.97). However, the average number of cigarettes smoked per day was low; only four women (two OC-users and two non-OC-users) reported smoking more than 10 cigarettes per day. Approximately 41% of participants consumed more than two cups of coffee per day, 22.7% consumed at least one cup of tea per day, and 17% reported using nutritional supplements, with no significant differences between OC-users and non-OC-users.

Table 2 summarises the blood biomarkers values in the three groups: all women, OC-users, and non-OC-users. Considering continuous values, OC-users had significantly lower TAC (*p* < 0.001), higher hydroperoxides (*p* < 0.001), higher transferrin (*p* < 0.001), lower transferrin saturation (*p* = 0.019), and higher hsCRP values (*p* < 0.001) than non-OC-users. Regarding categorical variables, OC-users were more likely than non-OC-users to have: low TAC (<1.07 FORD units; OR = 18.2), elevated/high hydroperoxides (≥300 FORT units; OR = 74.3; ≥330 FORT units; OR = 401, respectively), and an abnormal combined oxidative condition (defined by <1.07 FORD units combined with ≥300 FORT units; OR = 39.0). They were also more likely to have transferrin ≥ 330 mg dL^−1^ (OR = 9.74) and hsCRP ≥ 3 mg L^−1^ (OR = 11.1). Frequency of ID by ferritin < 12 µg L^−1^ or by sTfR-F index ≥ 1.5 did not differ between OC-users and non-OC-users.

Table 3 shows the comparison of the 71 women having an abnormal combined oxidative condition (i.e., combined low TAC < 1.07 FORD units and elevated OS ≥ 300 FORT units, of which 61 were OC-users and 10 non-OC-users), with 111 women (of which 15 OC-users and 96 non-OC-users) without this condition. Regarding continuous variables, women with an abnormal combined oxidative profile had: higher BMI (*p* = 0.009), higher cigarettes per day consumption (*p* = 0.001), higher WBC counts (*p* = 0.042), lower TAC (*p* < 0.001), higher OS hydroperoxides (*p* < 0.001), higher transferrin (*p* < 0.001), lower transferrin saturation (*p* = 0.002), and higher hsCRP (*p* < 0.001) values. Regarding categorical variables, these women were more likely to be: smokers (OR = 3.76), OC-users (OR = 39.0), with transferrin ≥ 330 mg dL^−1^ (OR = 6.58), and with hsCRP ≥ 3 mg L^−1^ (OR = 10.1). A trend toward higher BMI ≥ 23 kg m^−2^ was observed (OR = 2.39, *p* = 0.060^). Importantly, indicators of body iron status different from transferrin and transferrin saturation, including serum iron, ferritin, sTfR, sTfR-F index, in our study were not affected by the abnormal combined antioxidant defence/oxidative stress status. Of note, frequency of ID by ferritin < 12 µg L^−1^ or by sTfR-F index ≥ 1.5 did not differ between groups.

**Figure 1:**
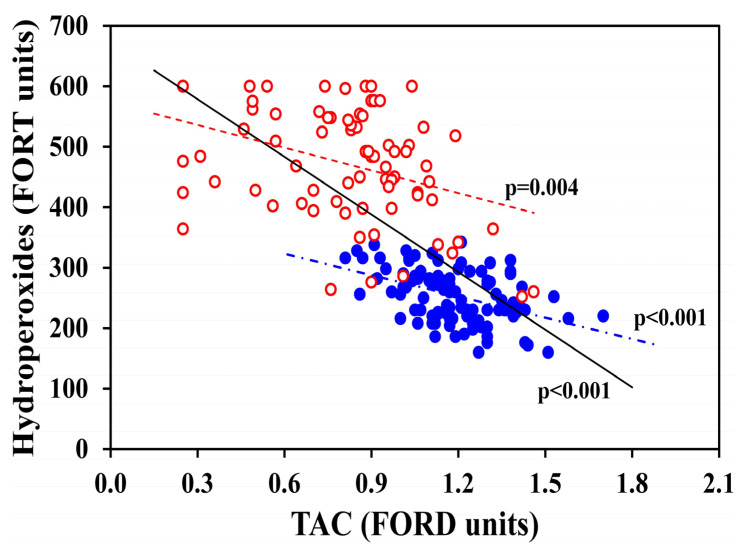
OS hydroperoxides (FORT units) are illustrated as a function of TAC (FORD units) in all 182 women, further distinguished in 76 OC-users (red empty dots) and 106 non-OC-users (blue full dots). The statistically significant inverse correlations between the two biomarkers are also shown: in the group of all women (*p* < 0.001, continuous black line); OC-users (*p* = 0.004, red dotted line); and non-OC-users (*p* < 0.001, blue point-dotted line).

**Figure 2:**
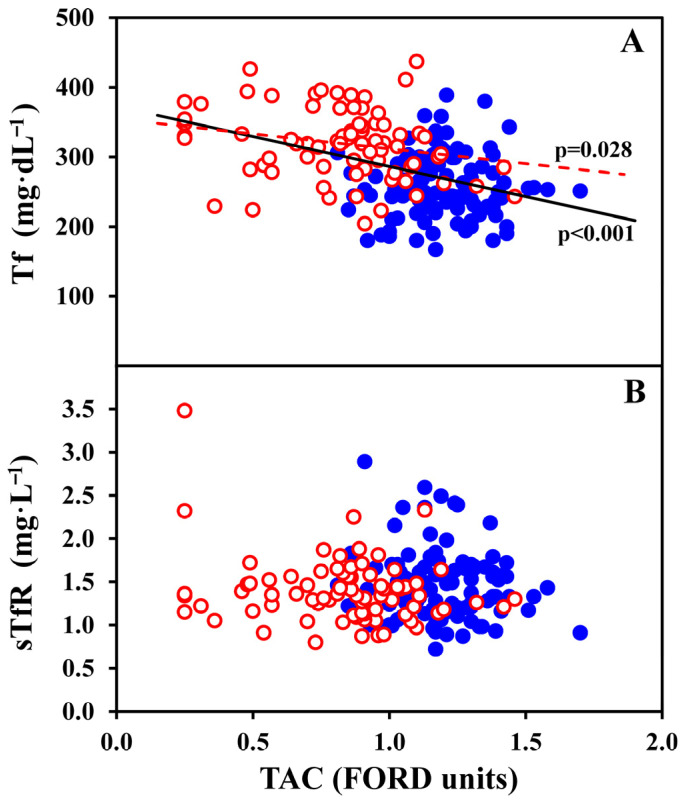
Tf (**A**) and sTfR (**B**) values are illustrated as a function of TAC (FORD units) in all 182 women, further distinguished in 76 OC-users (red empty dots) and 106 non-OC-users (blue full dots). The statistically significant correlations are also shown. Specifically, significant inverse correlations were observed for Tf in the group of all women (*p* < 0.001; continuous black line), and in OC-users (*p* = 0.028; red dotted line).

**Figure 3:**
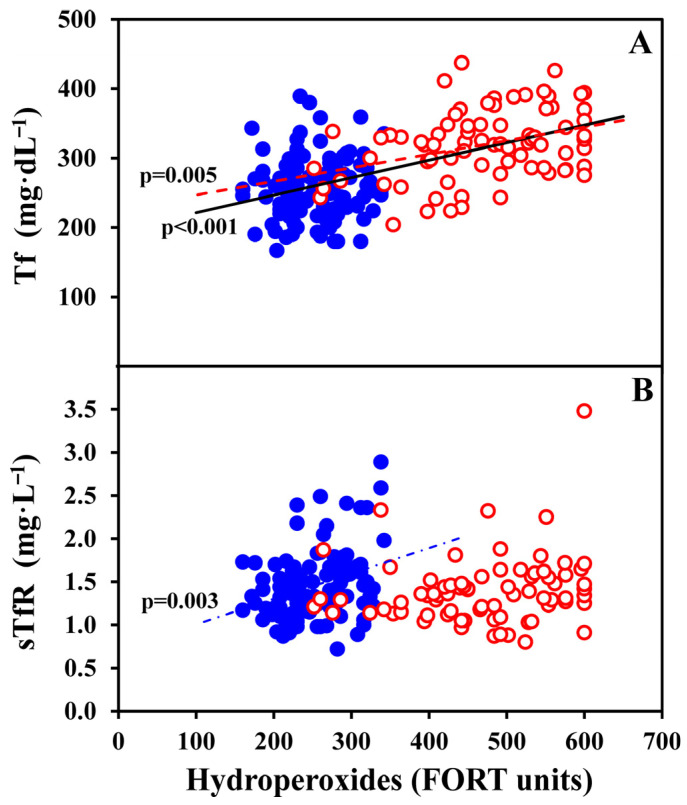
Tf (**A**) and sTfR (**B**) values are illustrated as a function of OS hydroperoxides (FORT units) in all 182 women, further distinguished in 76 OC-users (red empty dots) and 106 non-OC-users (blue full dots). The statistically significant positive correlations are also shown. In detail, Tf was significantly correlated to hydroperoxides in all women (*p* < 0.001; continuous black line), and in OC-users (*p* = 0.005; red dotted line); sTfR was significantly correlated to hydroperoxides only in non-OC-users (*p* = 0.003; blue point-dotted line).

Table 4 and Figure 1, Figure 2 and Figure 3 present the correlations of TAC (in FORD units) and OS hydroperoxides (in FORT units) with other study blood indicators, in all the 182 women, and, separately, in 76 OC-users and 106 non-OC-users. TAC was strongly inversely correlated with OS hydroperoxides in all women (*p* < 0.001), OC-users (*p* = 0.004) and non-OC-users (*p* < 0.001) as illustrated in Figure 1**,** which shows each individual value. Figure 2A shows individual data of TAC that were significantly correlated with transferrin in all women (*p* < 0.001) and OC-users (*p* = 0.028), while no significant relationships were observed for sTfR (Figure 2B). Furthermore, as reported in Table 4, TAC was significantly inversely correlated with hsCRP in all women (*p* < 0.001), OC-users (*p* = 0.012) and non-OC-users (*p* = 0.046), and with transferrin in all women (*p* < 0.001) and OC-users (*p* = 0.028), but not in non-OC-users. Lastly, TAC was mildly positively correlated with TfS% in all women (*p* = 0.044). No significant associations were found between TAC and sTfR, or sTfR-F index.

As further illustrated in Table 4, OS hydroperoxides (FORT units) showed a negative correlation with: Hb in non-OC-users (*p* = 0.045); Hct in all women (*p* = 0.044); and TfS% in all women (*p* < 0.001), OC-users (*p* = 0.001) and non-OC-users (*p* = 0.015). Conversely, OS hydroperoxides were positively correlated with WBC counts in all women (*p* = 0.011), and Tf in all women (*p* < 0.001) and OC-users (*p* = 0.005), as shown in detail in Figure 3A. Notably, OS hydroperoxides were markedly positively related with hsCRP in all women (*p* < 0.001), in OC-users (*p* < 0.001), and non-OC-users (*p* < 0.001). By contrast, no significant correlations were observed for OS hydroperoxides and sTfR in all women and OC-user, but a positive association was found in non-OC-users, as depicted in Figure 3B. In parallel, a similar profile was observed for the sTfR-F index (Table 4).

Table 5 illustrates the correlations of Tf and sTfR with other study biomarkers. Transferrin was positively correlated with RBC counts in all 182 women (*p* = 0.014) and OC-users (*p* = 0.013); and had strong positive relationships with sTfR and sTfR-F index in all the three groups of women. Transferrin was positively associated with hsCRP in all women (*p* < 0.001) and OC-users (*p* = 0.014). Conversely, as expected, Tf was negatively correlated to serum iron, TfS%, and ferritin in all the three groups of women. Finally, Tf was negatively associated with uric acid in OC-users only (*p* = 0.003).

Soluble transferrin receptor showed a mild negative correlation with Hb in non-OC-users (*p* = 0.044), and a positive correlation with RBC in OC-users (*p* = 0.032). Furthermore, consistently sTfR had negative correlations with serum iron, TfS%, and ferritin in all the three groups of women. As expected, sTfR was positively correlated with sTfR-F index in all the three groups (*p* < 0.001). Additionally, sTfR showed a positive correlation with folate in all women (*p* = 0.013), and non-OC-users (*p* = 0.025).

Finally, Table 6 shows the correlations of serum iron and ferritin with other study biomarkers. Serum iron was positively correlated to Hb in all 182 women (*p* = 0.021) and non-OC-users (*p* = 0.037), TfS% (*p* < 0.001 in the three groups of women), and ferritin in all women (*p* = 0.002) and non-OC-users (*p* = 0.003). Serum iron was negatively correlated to sTfR-F index in all the three groups of women, and to hsCRP in OC-users only (*p* = 0.017).

Ferritin was positively associated with: Hb in all women (*p* = 0.003) and non-OC-users (*p* = 0.011), Hct in all women, (*p* = 0.011), and TfS% in all women and non-OC-users (*p* < 0.001 for both groups of women). As expected, ferritin was negatively correlated to sTfR-F index (*p* < 0.001 in the three groups of women). Importantly, ferritin showed no association with TAC or OS hydroperoxides, supporting its independence from oxidative status. Additionally, ferritin had no associations with folate, hsCRP, uric acid, and creatinine.

**Table 1:** Demographic and behavioural characteristics of all 182 study women, and comparison between 76 OC-users and 106 non-OC-users. Results were presented as mean ± SD for continuous variables, and number (%), OR (95% CI) for dichotomous variables.

Characteristic	All Women (*n* = 182)	OC-Users (*n* = 76)	Non-OC-Users (*n* = 106)	*p*	OR (95% CI)
Age (years), mean ± SD	23.3 ± 4.3	23.4 ± 3.8	23.3 ± 4.6	0.231	
Weight (kg), mean ± SD	59.4 ± 7.8	60.4 ± 7.3	58.6 ± 8.1	0.094	
Height (m), mean ± SD	168.7 ± 6.4	169.0 ± 6.2	168.5 ± 6.6	0.515	
BMI (kg m^−2^), mean ± SD ^a^	20.8 ± 2.1	21.1 ± 2.1	20.6 ± 2.0	0.149	
BMI ≥ 23 kg m^−2 a^	22 (12.2)	10 (13.2)	12 (11.5)	0.743	1.16 (0.47–2.85)
University education, *n* (%)	144 (79.1)	62 (81.6)	82 (77.4)	0.490	1.30 (0.62–2.71)
Unmarried, *n* (%)	170 (93.4)	72 (94.7)	98 (92.5)	0.540	1.47 (0.43–5.07)
Nulliparity, *n* (%)	178 (97.8)	74 (97.4)	104 (98.1)	1.000	0.71 (0.10–5.17)
Smokers, *n* (%) ^b^	40 (22.7)	25 (34.2)	15 (14.4)	**0.003**	**3.06 (1.47–6.34)**
Cigarettes day^−1^, mean ± SD ^b^	1.1 ± 2.9	1.4 ± 2.6	0.9 ± 3.2	**0.004**	
Cigarettes day^−1^ in smokers, mean ± SD ^b^	4.8 ± 4.6	4.0 ± 3.0	6.1 ± 6.3	0.208	
>5 cigarettes day^−1^, *n* (%) ^b^	17 (9.7)	9 (12.3)	8 (7.8)	0.317	1.67 (0.61–4.56)
>10 cigarettes day^−1^, *n* (%) ^b^	4 (2.3)	2 (2.7)	2 (1.9)	0.728	1.42 (0.20–10.3)
>2 coffee cups day^−1^, *n* (%) ^b,c^	72 (40.9)	29 (39.7)	43 (41.7)	0.788	0.92 (0.50–1.69)
≥1 tea cups day^−1^, *n* (%) ^b^	40 (22.7)	19 (26.0)	21 (20.4)	0.380	1.37 (0.68–2.79)
Nutritional supplement use, *n* (%) ^b^	30 (17.0)	14 (19.2)	16 (15.5)	0.527	1.29 (0.59–2.84)

Comparison of OC-users with non-OC-users was performed by two-tailed Mann–Whitney *p* value for continuous variables, and with *p* value evaluated by chi-square Fisher or Pearson test, as was appropriate, for dichotomous variables. Significant *p* values and OR were indicated in bold. ^a^ BMI data were available for 104 out of 106 non-OC-users. ^b^ Data were available for total 176 women, of which 73 out of 76 OC-users, and 103 out of 106 non-OC-users. ^c^ Italian espresso coffee cups.

**Table 2:** Venous blood biomarkers of all 182 study women, and comparison between 76 OC-users and 106 non-OC-users. Results were presented as mean ± SD or median (IQR), as was appropriate for continuous variables, and number (%), OR (95% CI) for dichotomous variables.

Characteristic	All Women (*n* = 182)	OC-Users (*n* = 76)	Non-OC-Users (*n* = 106)	*p*	OR (95% CI)
Hb (g L^−1^)	12.8 ± 0.8	12.8 ± 0.7	12.8 ± 0.85	0.785	
Hct (%)	38.1 ± 2.4	37.9 ± 2.2	38.2 ± 2.6	0.389	
RBC (10^12^ L^−1^)	4.35 ± 0.34	4.33 ± 0.29	4.36 ± 0.38	0.754	
WBC (10^9^ L^−1^)	6.18 (5.24–7.18)	6.47 (5.53–7.35)	5.92 (5.06–7.01)	0.124	
TAC, FORD units	1.07 (0.89–1.22)	0.87 (0.70–0.98)	1.17 (1.07–1.30)	**<0.001**	
TAC ≥ 1.07 FORD units	94 (51.6)	12 (15.8)	82 (77.4)	**<0.001**	**0.05 (0.03–0.12)**
TAC < 1.07 FORD units	88 (48.4)	64 (84.2)	24 (22.6)	**<0.001**	**18.2 (8.47–39.2)**
OS hydroperoxides, FORT units	294 (234–445)	480 (410–548)	254 (220–287)	**<0.001**	
OS hydroperoxides, ≥300 FORT units	88 (48.4)	71 (93.4)	17 (16.0)	**<0.001**	**74.3 (26.2–211.3)**
TAC < 1.07 FORD and OS ≥ 300 FORT units	71 (39.0)	61 (80.3)	10 (9.4)	**<0.001**	**39.0 (16.5–92.5)**
Hydroperoxides, ≥330 FORT units	73 (40.1)	70 (92.1)	3 (2.8)	**<0.001**	**401 (97–1655)**
TAC < 1.07 FORD and OS ≥ 330 FORT units	62 (34.1)	61 (80.3)	1 (0.01)	**<0.001**	**427 (55–3312)**
Serum iron μg dL^−1^	88 (64–118)	90 (65–120)	86 (63–117)	0.439	
Tf mg dL^−1^	277 (243–323)	321 (285–348)	252 (226–286)	**<0.001**	
Tf ≥ 330 mg dL^−1^	38 (2.1)	31 (40.8)	7 (6.6)	**<0.001**	**9.74 (3.99–23.8)**
TfS %	22.2 (15.6–31.8)	19.8 (14.7–27.3)	26.6 (16.2–32.9)	**0.019**	
TfS < 18%	61 (33.5)	30 (39.5)	31 (29.2)	0.149	1.58 (0.85–2.94)
TfS < 15%	40 (22.0)	20 (26.3)	20 (18.9)	0.231	1.54 (0.76–3.11)
Ferritin μg L^−1^	19 (11–34)	17 (11–33)	19 (11–35)	0.489	
Ferritin < 12 μg L^−1^	53 (29.1)	22 (28.9)	31 (29.2)	0.965	0.99 (0.51–1.89)
sTfR mg L^−1^	1.36 (1.18–1.61)	1.34 (1.15–1.56)	1.41 (1.19–1.66)	0.236	
sTfR ≥ 1.76 mg L^−1^	23 (12.6)	8 (10.5)	15 (14.2)	0.468	0.71 (0.29–1.78)
sTfR-F index	1.36 (1.18–1.61)	1.05 (0.79–1.45)	1.08 (0.78–1.49)	0.834	
sTfR-F index ≥ 1.5	46 (25.3)	19 (25.0)	27 (25.5)	0.942	0.97 (0.49–1.92)
Folate μg L^−1^	4.6 (3.5–6.3)	4.6 (3.5–6.1)	4.5 (3.6–6.4)	0.743	
hsCRP mg L^−1^	0.51 (0.21–1.51)	1.46 (0.80–4.18)	0.27 (0.10–0.51)	**<0.001**	
hsCRP ≥ 3.0 mg L^−1^	32 (17.6)	27 (35.5)	5 (4.7)	**<0.001**	**11.1 (4.04–30.7)**
Uric acid mg L^−1^	4.1 (3.5–4.5)	4.0 (3.5–4.4)	4.1 (3.5–4.6)	0.427	
Creatinine mg L^−1^	0.93 ± 0.13	0.94 ± 0.14	0.92 ± 0.13	0.179	

Comparison of OC-users with non-OC-users was performed by two-tailed Mann–Whitney *p* value for continuous variables, and with *p* value evaluated by a chi-square, Fisher or Pearson test, as was appropriate, for dichotomous variables. Significant *p* values and OR were indicated in bold.

**Table 3:** Comparison of demographic/behavioural characteristics and venous blood biomarkers of 71 women with combined abnormal low total antioxidant capacity (TAC) and elevated oxidative stress (OS) profile defined by TAC < 1.07 FORD and OS hydroperoxides ≥ 300 FORT units with 111 women not having this condition. Results were presented as mean ± SD or median (IQR as was appropriate for continuous variables, and number (%), OR (95% CI) for dichotomous variables.

Characteristic	Low TAC and Elevated OS (*n* = 71)	Non-Abnormal TAC and OS (*n* = 111)	*p*	OR (95% CI)
Age (years), mean ± SD	23.7 ± 4.1	23.1 ± 4.7	0.097	
Weight (kg), mean ± SD ^a^	61.0 ± 8.3	58.4 ± 7.3	0.081	
Height (m), mean ± SD	168.6 ± 6.8	168.8 ± 6.2	0.674	
BMI (kg m^−2^), mean ± SD ^a^	21.4 ± 2.2	20.4 ± 1.9	**0.009**	
BMI ≥ 23 kg m^−2 a^	13 (18.3)	9 (8.3)	0.060 ^	2.39 (0.96–5.94)
University education, *n* (%)	55 (77.5)	89 (80.2)	0.660	0.85 (0.41–1.76)
Unmarried, *n* (%)	65 (91.5)	105 (94.6)	0.542	0.62 (0.19–2.00)
Nulliparity, *n* (%)	69 (97.2)	109 (98.2)	0.644	0.63 (0.09–4.60)
Smokers, *n* (%) ^b^	26 (36.6)	14 (13.3)	**<0.001**	**3.76 (1.79–7.88)**
Cigarettes day^−1^, mean ± SD ^b^	1.5 ± 2.6	0.8 ± 3.1	**0.001**	
Cigarettes day^−1^ in smokers, mean ± SD ^b^	4.1 ± 2.8	6.0 ± 6.7	0.452	
>5 cigarettes day^−1^, *n* (%) ^b^	10 (14.1)	7 (6.7)	0.110	2.30 (0.83–6.35)
>10 cigarettes day^−1^, *n* (%) ^b^	2 (2.8)	2 (1.9)	0.692	1.49 (0.21–10.9)
>2 coffee cups day^−1^, *n* (%) ^b,c^	31 (43.7)	41 (39.0)	0.541	1.21 (0.66–2.23)
Nutritional supplement use, *n* (%) ^b^	13 (18.3)	17 (16.2)	0.714	1.16 (0.52–2.57)
Hb (g L^−1^)	12. 7 ± 0.8	12.8 ± 0.8	0.706	
Hct (%)	37.8 ± 2.2	38.2 ± 2.5	0.181	
RBC (10^12^ L^−1^)	4.34 ± 0.29	4.35 ± 0.37	0.855	
WBC (10^9^ L^−1^)	6.49 (5.58–7.39)	5.88 (5.05–6.97)	**0.042**	
TAC, FORD units	0.86 (0.66–0.95)	1.18 (1.11–1.30)	**<0.001**	
TAC ≥ 1.07 FORD units	0 (0)	94 (84.7)	**<0.001**	**0.15 (0.10–0.24)**
Low TAC < 1.07 FORD units	71 (100)	17 (15.3)	**<0.001**	**0.15 (0.10–0.24)**
OS hydroperoxides, FORT units	484 (402–551)	256 (220–286)	**<0.001**	
OS hydroperoxides, ≥300 FORT units	71 (100)	17 (15.3)	**<0.001**	**0.15 (0.10–0.24)**
OC-user	61 (85.9)	15 (13.5)	**<0.001**	**39.0 (16.5–92.5)**
Serum iron μg dL^−1^	86 (65–114)	89 (63–123)	0.635	
Tf mg dL^−1^	319 (282–348)	257 (229–293)	**<0.001**	
Tf ≥ 330 mg dL^−1^	28 (39.4)	10 (9.0)	**<0.001**	**6.58 (2.94–14.7)**
TfS %	19.1 (14.8–24.7)	26.9 (15.6–34.6)	**0.002**	
TfS < 18%	29 (40.8)	32 (28.8)	0.094	1.70 (0.91–3.19)
TfS < 15%	18 (25.4)	22 (19.8)	0.379	1.37 (0.68–2.79)
Ferritin μg L^−1^	17 (11–36)	19 (11–33)	0.487	
Ferritin < 12 μg L^−1^	21 (29.6)	32 (28.8)	0.914	1.04 (0.54–1.99)
sTfR mg L^−1^	1.36 (1.16–1.56)	1.36 (1.18–1.65)	0.753	
sTfR ≥ 1.76 mg L^−1^	8 (11.3)	15 (13.5)	0.656	0.81 (0.32–2.03)
sTfR-F index	1.06 (0.77–1.50)	1.07 (0.80–1.47)	0.804	
sTfR-F index ≥ 1.5	19 (26.8)	27 (24.3)	0.712	1.14 (0.57–2.25)
Folate μg L^−1^	4.6 (3.5–6.3)	4.5 (3.5–6.4)	0.669	
hsCRP mg L^−1^	1.5 (0.7–4.9)	0.3 (0.1–0.6)	**<0.001**	
hsCRP ≥ 3.0 mg L^−1^	26 (36.6)	6 (5.4)	**<0.001**	**10.1 (3.89–26.3)**
Uric acid mg L^−1^	3.8 (3.2–4.5)	4.1 (3.6–4.6)	0.122	
Creatinine mg L^−1^	0.94 ± 0.13	0.92 ± 0.13	0.334	

Comparison of women with and without a combined abnormal low TAC/elevated OS condition was performed by two-tailed Mann–Whitney *p* value for continuous variables, and with *p* value evaluated by a chi-square, Fisher or Pearson test, as was appropriate, for dichotomous variables. Significant *p* values and OR were indicated in bold. ^ Indicated a trend. ^a^ Weight and BMI data were available for 109 out of 111 women with non-abnormal TAC and OS. ^b^ Data were available for 105 out of 111 women with non-abnormal combined low TAC/elevated OS. ^c^ Italian espresso coffee cups.

**Table 4:** Correlation of total antioxidant capacity (TAC, in FORD units), oxidative stress (OS hydroperoxides, in FORT units), with serum iron status biomarkers in all women (*n* = 182), OC-users (*n* = 76), and non-OC-users (*n* = 106), by Spearman two-tailed Rho coefficient r_s_.

Measure	TAC, FORD Units All Women (*n* = 182)	TAC, FORD Units OC-Users (*n* = 76)	TAC, FORD Units Non-OC-Users (*n* = 106)	OS, FORT Units All Women (*n* = 182)	OS, FORT Units OC-Users (*n* = 76)	OS. FORT Units Non-OC-Users (*n* = 106)
OS, FORT units	**−0.732** ***p* < 0.001**	**−0.328** ***p* = 0.004**	**−0.410** ***p* < 0.001**	-	-	-
Hb g L^−1^	−0.014 NS	0.035 NS	−0.069 NS	−0.089 NS	−0.175 NS	**−0.195** ***p* = 0.045**
Hct (%)	0.057 NS	0.138 NS	−0.044 NS	**−0.149** ***p* = 0.044**	−0.143 NS	−0.175 NS
RBC (10^12^ L^−1^)	−0.016 NS	−0.019 NS	−0.064 NS	−0.040 NS	−0.003 NS	−0.026 NS
WBC (10^9^ L^−1^)	−0.130 *p* = 0.079 ^	−0.039 NS	−0.115 NS	**0.189** ***p* = 0.011**	0.116 NS	0.178 *p* = 0.068 ^
Serum iron μg dL^−1^	−0.047 NS	0.001 NS	−0.063 NS	−0.106 NS	−0.108 NS	−0.104 NS
Tf mg dL^−1^	**−0.428** ***p* < 0.001**	**−0.253** ***p* = 0.028**	−0.001 NS	**0.534** ***p* < 0.001**	**0.317** ***p* = 0.005**	0.080 NS
TfS %	**0.150** ***p* = 0.044**	0.086 NS	−0.068 NS	**−0.312** ***p* < 0.001**	**−0.359** ***p* = 0.001**	**−0.235** ***p* = 0.015**
Ferritin μg L^−1^	−0.037 NS	0.010 NS	−0.168 *p =* 0.085 ^	−0.097 NS	−0.121 NS	−0.113 NS
sTfR mg L^−1^	−0.004 NS	−0.138 NS	−0.057 NS	0.069 NS	0.190 NS	**0.290** ***p* = 0.003**
sTfR-F index	0.024 NS	−0.053 NS	0.054 NS	0.089 NS	0.149 NS	**0.229** ***p* = 0.018**
Folate μg L^−1^	−0.068 NS	−0.044 NS	−0.144 NS	−0.038 NS	0.111 NS	−0.081 NS
hsCRP mg L^−1^	**−0.581** ***p* < 0.001**	**−0.287** ***p* = 0.012**	**−0.194** ***p* = 0.046**	**0.695** ***p* < 0.001**	**0.392** ***p* < 0.001**	**0.411** ***p* < 0.001**
Uric acid mg L^−1^	0.083 NS	−0.045 NS	0.058 NS	−0.12 *p* = 0.097 ^	−0.158 NS	−0.068 NS
Creatinine mg L^−1^	−0.016 NS	0.103 NS	0.121 NS	0.065 NS	−0.005 NS	−0.034 NS

Statistical significant r_s_ and *p* values were indicated in bold. ^ Indicated a trend because the *p* value was between ≥0.050 and <0.100. NS indicated non significant *p* value ≥ 0.100.

**Table 5:** Correlation of transferrin and soluble transferrin receptor (sTfR) with other serum biomarkers in all women (*n* = 182), OC-users (*n* = 76), and non-OC-users (*n* = 106), by Spearman’s two-tailed Rho coefficient r_s_.

Measure	Transferrin All Women (*n* = 182)	Transferrin OC-Users (*n* = 76)	Transferrin Non-OC-Users (*n* = 106)	sTfR All Women (*n* = 182)	sTfR OC-Users (*n* = 76)	sTfR Non-OC-Users (*n* = 106)
Hb g L^−1^	0.079 NS	0.155 NS	0.046 NS	−0.134 *p* = 0.071 ^	−0.047 NS	**−0.196** ***p* = 0.044**
Hct (%)	0.042 NS	0.120 NS	0.084 NS	−0.029 NS	0.088 NS	−0.120 NS
RBC (10^12^ L^−1^)	**0.182** ***p* = 0.014**	**0.283** ***p* = 0.013**	0.162 NS	0.109 NS	**0.247** ***p* = 0.032**	0.002 NS
WBC (10^9^ L^−1^)	0.109 NS	0.036 NS	0.051 NS	−0.036 NS	0.003 NS	−0.036 NS
Serum iron μg dL^−1^	**−0.172** ***p* = 0.020**	**−0.231** ***p* = 0.045**	**−0.255** ***p* = 0.008**	**−0.264** ***p* < 0.001**	**−0.298** ***p* = 0.009**	**−0.244** ***p* = 0.012**
TfS %	**−0.538** ***p* < 0.001**	**−0.493** ***p* < 0.001**	**−0.526** ***p* < 0.001**	**−0.334** ***p* < 0.001**	**−0.372** ***p* = 0.001**	**−0.340** ***p* < 0.001**
Ferritin μg L^−1^	**−0.384** ***p* < 0.001**	**−0.297** ***p* = 0.009**	**−0.533** ***p* < 0.001**	**−0.503** ***p* < 0.001**	**−0.519** ***p* < 0.001**	**−0.502** ***p* < 0.001**
sTfR mg L^−1^	**0.308** ***p* < 0.001**	**0.367** ***p* = 0.001**	**0.471** ***p* < 0.001**	-	-	-
sTfR-F index	**0.390** ***p* < 0.001**	**0.360** ***p* = 0.001**	**0.571** ***p* < 0.001**	**0.842** ***p* < 0.001**	**0.825** ***p* < 0.001**	**0.853** ***p* < 0.001**
Folate μg L^−1^	0.031 NS	−0.162 NS	**0.200** ***p* = 0.040**	**0.184** ***p* = 0.013**	0.141 NS	**0.217** ***p* = 0.025**
hsCRP mg L^−1^	**0.391** ***p* < 0.001**	**0.282** ***p* = 0.014**	−0.111 NS	−0.049 NS	0.064 NS	−0.002 NS
Uric acid mg L^−1^	−0.067 NS	**−0.349** ***p* = 0.003**	0.146 NS	0.073 NS	−0.016 NS	0.123 NS
Creatinine mg L^−1^	0.011 NS	−0.151 NS	−0.015 NS	−0.014 NS	0.071 NS	−0.062 NS

Statistical significant r_s_ and *p* values were indicated in bold. ^ Indicated a trend because the *p* value was between ≥0.050 and <0.100. NS indicated non significant *p* value ≥ 0.100.

**Table 6:** Correlation of serum iron and ferritin with other serum biomarkers in all women (*n* = 182), OC-users (*n* = 76), and non-OC-users (*n* = 106), by Spearman’s two-tailed Rho coefficient r_s_.

Measure	Serum Iron All Women (*n* = 182)	Serum Iron OC-Users (*n* = 76)	Serum Iron Non-OC-Users (*n* = 106)	Ferritin All Women (*n* = 182)	Ferritin OC-Users (*n* = 76)	Ferritin Non-OC-Users (*n* = 106)
Hb g L^−1^	**0.171** ***p* = 0.021**	0.131 NS	**0.203** ***p* = 0.037**	**0.219** ***p* = 0.003**	0.186 NS	**0.247** ***p* = 0.011**
Hct (%)	0.105 NS	0.078 NS	0.128 NS	**0.187** ***p* = 0.011**	0.196 NS	0.187 *p* = 0.055 ^
RBC (10^12^ L^−1^)	−0.029 NS	−0.163 NS	0.090 NS	0.036 NS	0.000 NS	0.082 NS
WBC (10^9^ L^−1^)	0.032 NS	−0.033 NS	0.067 NS	0.099 NS	0.115 NS	0.109 NS
TfS %	**0.911** ***p* < 0.001**	**0.942** ***p* < 0.001**	**0.945** ***p* < 0.001**	**0.334** ***p* < 0.001**	0.218 *p =* 0.058 ^	**0.415** ***p* < 0.001**
Ferritin μg L^−1^	**0.228** ***p* = 0.002**	0.153 NS	**0.288** ***p* = 0.003**	**-**	-	**-**
sTfR-F index	**−0.266** ***p* < 0.001**	**−0.243** ***p* = 0.034**	**−0.282** ***p* = 0.003**	**−0.874** ***p* < 0.001**	**−0.894** ***p* < 0.001**	**−0.863** ***p* < 0.001**
Folate μg L^−1^	−0.027 NS	0.003 NS	−0.043 NS	−0.092 NS	−0.042 NS	−0.123 NS
hsCRP mg L^−1^	−0.103 NS	**−0.273** ***p* = 0.017**	−0.124 NS	0.039 NS	0.038 NS	0.083 NS
Uric acid mg L^−1^	−0.076 NS	−0.020 NS	−0.104 NS	0.064 NS	0.176 NS	−0.031 NS
Creatinine mg L^−1^	−0.050 NS	−0.060 NS	−0.073 NS	0.032 NS	−0.023 NS	0.066 NS

Statistical significant r_s_ and *p* values were indicated in bold. ^ Indicated a trend because the *p* value was between ≥0.050 and <0.100. NS indicated non significant *p* value ≥ 0.100.

## 4. Discussion

To our knowledge, this is the first study to simultaneously examine in Caucasian young women: total antioxidant capacity, oxidative stress (as lipid hydroperoxides), inflammation (as hsCRP), iron status biomarkers, and effects of oral contraception.

Our study subjects consisted of young (mean age 23 years), healthy women, a population in which early and effective prevention strategies against future diseases, especially cardiovascular diseases and tumours, are potentially feasible and effective [49,50,51,52,53,54,55,56,57].

### 4.1. Total Antioxidant Defence Capacity (TAC by FORD Units)

We observed that only approximately half (51.6%) of participants had optimal TAC values (≥1.07 FORD units), but with a marked disparity according to OC use, as much as 77.4% of non-OC-users versus 15.8% of OC-users had normal TAC values (*p* < 0.001). These findings reveal a strong unexpected impact of OC use in dampening systemic total antioxidant defence capacity. Similarly, approximately half of the women (48.4%) exhibited elevated OS lipid hydroperoxides above the normal values (≥300 FORT units), again with a pronounced difference according to OC use, 93.4% of OC-users versus 16.0% of non-OC-users (OR = 74.3, *p* < 0.001). This difference was even more impressive by use of the FORT ≥ 330 units cutoff, a level indicating overt strong oxidative stress (92.1% OC-users versus 2.8% non-OC-users).

In this study, TAC values were strongly inversely associated with oxidative stress, confirming previous observations in female athletes OC-users [15]. Importantly, in the present study, this inverse relationship was observed consistently across all groups of healthy young women, including both OC-users, and non-OC-users. However, the directionality of this relationship cannot be determined from the present cross-sectional design; it remains unclear whether reduced antioxidant capacity leads to increased oxidative stress or whether vice versa high oxidative stress depletes antioxidant reserves, and/or whether both mechanisms may coexist. Future enlarged studies are needed to assess by which biological pathways OC use could depress TAC in the vast majority of OC-users, and which component (or components) of OC formulations might specifically reduce TAC, directly or indirectly.

One of the main new findings of our study was that in overall healthy women, TAC was strongly inversely associated with hsCRP, indicating a close link between antioxidant capacity and low-grade inflammation. This inverse association was observed in all groups of women, suggesting that this interplay may reflect general, fundamental physiological mechanisms rather than being solely attributed to hormonal exposure. We cannot, however, infer whether low TAC makes hsCRP to increase or vice versa the increase in inflammation causes reduction in the TAC defence, or if both may contribute. This may be crucial to be determined especially for the interpretation of OC use effects, since both low TAC and elevated inflammation resulted much more frequent in OC-users in respect to non-OC-users.

The present study found that oxidative stress (hydroperoxides) was associated with increased inflammation (as assessed by hsCRP) confirming previous investigations performed in asymptomatic women [16], and in many pathologic conditions like hypertension, atherosclerosis, metabolic syndrome, type 1 and type 2 diabetes, liver diseases, and tumours [14,55,56,57,58,59], supporting the concept that oxidative stress and inflammation are tightly interconnected processes. It is to highlight that, our study is observational and cross-sectional; thus, it does not allow us to determine causal relationships, i.e., whether the increase in oxidative stress associated with the dampening of the antioxidant defence is the driver of the increased inflammation or the contrary, or likely the effects are bidirectional, thus creating an auto-sustaining loop. Evidence points to oxidative stress as a major trigger of increased inflammation [11], particularly via activation of the nuclear factor ĸB (NF-ĸB), a transcription factor, which is a pivotal component of the inflammatory response [56,60,61]. On the other hand, inflammatory processes may enhance ROS production, for instance, via enzymatic systems such as the NADPH oxidase complex of phagocytes, which is known to generate the aggressive unstable superoxide anion radical, a precursor of H_2_O_2_. In turn, H_2_O_2_ can modulate the activity of redox-responsive transcriptional factors like NF-ĸB, nuclear factor erythroid 2-related factor 2 (NRF2), and hypoxia inducible factor-1 (HIF-1), also mediating redox-based epigenetic modifications [61]. Overall, survival and function of immune cells are under redox control, and depend on intracellular/extracellular levels of ROS and reactive nitrogen species (RNS). Thus, redox factors are involved in the activation of immune response, specifically ROS are responsible for oxidative modification of proteins in macrophage polarisation and neutrophil functions [61].

Overall, our findings suggest that TAC, oxidative stress, and inflammation constitute an interconnected biological triad, strongly modulated by OC use.

A further observation is that we first found that TAC was inversely related to a commonly used serum biomarker of iron status, transferrin, in all women and in OC-users, but not in non-OC-users.

### 4.2. Abnormal Combined Low TAC and Elevated Oxidative Stress Condition (Namely, FORD < 1.07 Associated with FORT ≥ 300 Units)

The abnormal combined condition of low TAC and elevated OS was more frequent in OC-users (OR = 39.0), smokers (OR = 3.76), and tended to be correlated with elevated BMI ≥ 23 kg m^−2^ (OR = 2.39). Thus, OC use resulted to be the main driver of this adverse combined condition in our population of young women. Regarding smoking effects, it is well known that smoking is an inducer of oxidative stress [62]; however, it was unexpected that even a low number of cigarettes per day (on average 1.5 versus 0.8 cigarettes per day) can be a risk factor for low TAC/increased OS. The present findings concur with a recent study [50], that measured TAC and total OS in the serum of university students, demonstrating almost doubly reduced TAC and increased OS in smokers compared to non-smokers, and the same trend was observed for passive smokers although with a lower magnitude [50,51]. Overall, these observations sustain intervention strategies to reduce smoking in young people [50,51].

Regarding BMI effects on the combined low TAC/increased OS condition, there is consolidated evidence that increased BMI and particularly obesity is associated with oxidative stress [51,63]. Interestingly, however, we found that, adiposity, even without obesity, in overweight women by BMI ≥ 23 kg m^−2^ tended to be a risk factor, confirming this cutoff as a potentially risky condition [48].

Additionally, the abnormal combined TAC/OS condition was associated with 5-fold higher continuous values of hsCRP, and 10-fold more frequent levels of hsCRP ≥ 3 mg L^−1^, which are considered inflammation values at risk for future cardiovascular events [45]. It is to remark that, as recently claimed, reduction in hsCRP already in young age is an important goal of strategies aimed at prevention of cardiovascular diseases in women [49].

Moreover, our results showed that, among the iron status biomarkers, the abnormal condition consisting of combined low TAC and elevated OS was associated with higher transferrin and lower transferrin saturation, likely a consequence of the inverse relationship of transferrin to TAC (above described). It is likely that some specific effects modulate blood concentration of transferrin, a protein produced mainly by the liver [64]. Conversely, no other iron biomarker, including serum iron, ferritin, sTfR, and sTfR-F-index was modulated by the abnormal combined TAC/OS condition. In other words, iron homeostasis does not appear to be altered by the abnormal TAC/OS status found in healthy young women.

It has to be mentioned that the FORD assay evaluated the total blood antioxidant defence capacity including several antioxidant molecules and enzymatic activities, so that we cannot attest which specific component (or components) of the antioxidant defence was dampened in women with the low TAC condition. However, we measured uric acid, that is considered a serum antioxidant, and which was not significantly different between women with the abnormal combined low TAC/elevated OS condition and the remaining women. By contrast, in a study including type 1 diabetes patients a negative correlation of oxidative stress with uric acid was observed [14].

Regarding TAC/OS status, roles of reduced thiols were described by some studies. Specifically, an investigation showed that OC use causes a significant increase in plasma peroxides and decrease in reduced thiols [17]. This observation is of interest because reduced levels of serum free thiols have been significantly associated with the female risk of cardiovascular events [65]. Additionally, a study showed that the oxidised form of cysteine, namely cystine (which represent the major serum extracellular thiol/disulfide redox control system) was associated with increase in the proinflammatory cytokine IL-1beta in human plasma [66], thus, demonstrating an interplay between oxidised thiols and inflammation. Of interest, a Belgian study [67] performed on 40–48 years old women, observed a significant increase in blood lipid peroxides, copper, copper to zinc ratio, and selenium, and lower levels of beta-carotene and gamma-tocopherol among OC-users compared to non-OC-users. Conversely, blood concentrations of vitamin C, alpha-tocopherol and zinc were unaffected by OC use. Additionally, the level of thiol proteins was lower for OC-users than for women with IUDs. Of relevance, a strong positive correlation between the concentration of plasma copper and lipid peroxides was found for all study women [67].

It remains to be verified whether the increased risk of cardiovascular events, particularly thromboembolism [68,69,70], myocardial infarction, and stroke associated with OC use [70,71] could be mediated, at least in part, by the prolonged decrease in TAC, and/or increased OS, in addition to the increase in chronic low-grade inflammation [72,73]. Detailed subject-specific prospective studies should be necessary.

### 4.3. Iron Deficiency (ID)

In the present study, we found iron deficiency (ID), as evaluated by ferritin < 12 µg L^−1^, in 29.1% of women, without significant difference between OC-users (28.9%) and non-OC-users (29.2%), roughly in line with previous investigations performed in pre-menopausal Caucasian Italian women, which assessed depleted iron stores in approximately one-third of fertile age females [30,37,38], mainly due to insufficient iron intake [29,30,31,35]. In our study ID was not modulated by the combined oxidative condition consisting of low TAC with elevated OS, suggesting that oxidative imbalance does not substantially alter iron homeostasis in this population.

ID occurs frequently in premenopausal women and clinical management of ID is recognised as challenging by practitioners, because this condition can be asymptomatic or associated with some generic symptoms like fatigue, reduced concentration, depression, pallor, headache, and others [27,28,29,30,31,74]. Of note, assessment of iron status in healthy individuals is important pre- and post-donation of blood [27,29,33,35]. Thus, the use of objective ID biomarkers not affected by different conditions like inflammation and/or oxidative stress or drugs commonly used as OC is of crucial importance for the clinical practice [29,30,31,32,33,34,35].

In the present investigation, we explored the complex relationships of iron homeostasis, antioxidant defence, oxidative stress, inflammation, and OC use affecting ID biomarkers. We found that in non-pathological fertile-age women, among ID biomarkers, ferritin was unaffected by OC use, antioxidant defence, oxidative stress status, and low-grade inflammation.

### 4.4. Transferrin and Soluble Transferrin Receptor (sTfR)

Transferrin is the main protein that transports iron in the plasma, so that iron bound to Tf can be supplied to tissues via binding to the transferring receptor (TfR) present in cells, mainly of the erythropoietic system [27,28,36]. It is generally recognised that the current gold standard iron store marker is serum ferritin; however, many clinicians continue to rely on measurement of serum iron, transferrin, and transferrin saturation to assess iron status in women [27,28,30], although investigations showed that transferrin levels are highly increased by OC-use [37,64,75]. In the present study, we confirm elevation of Tf in OC-users. The reasons for such an increase in Tf have been partially explained by the effects of oestrogen, which stimulates the liver to produce more proteins, including transferrin [37,64,75]; nevertheless, previous studies did not evaluate whether antioxidant defence and oxidative stress were implicated in this phenomenon [37,64].

This work is the first to show that antioxidant defence/oxidative stress status have a major impact in modulating serum Tf concentrations in all women and OC-users. Additionally, we found that transferrin was inversely related to uric acid in OC-users. Overall, these findings are intriguing also because transferrin is a negative acute phase element [27,36,64].

In our present study, we found that sTfR (i.e., the circulating truncated form of the cell membrane TfR) and the sTfR-F index were not affected by TAC values irrespective of OC use, and they were not associated with OS hydroperoxides in all women and in OC-users, but had a positive correlation with OS in non-OC-users. Such last finding was unexpected, and might be explained by the block of membrane bound receptor recycling caused by oxidative stress [76], which is associated with receptor redistribution but not to receptor loss, thus likely inducing an increase in the circulating form of TfR. This biological pathway was interpreted as a physiological protective mechanism reducing the pro-oxidant iron uptake by the cells in the presence of oxidative stress [76]. In addition, oxidative stress might contribute to modulate iron-regulatory RNA-binding proteins (IRPs), that, by binding to iron-responsive elements (IREs) of TfR mRNA, may regulate sTfR concentrations [77]. Our present findings are partly in contrast with a study performed in 117 Latin-American premenopausal and postmenopausal women with a mean age of 46 years (20- to 65-year-olds), in which sTfR was found positively correlated with TAC [78]. However, that study failed to evaluate oxidative stress and use of external hormone treatments by women.

Recent evidence suggests that sTfR has pleiotropic effects, in addition to reflect erythropoietic activity and iron balance [76]. In our study sTfR was positively correlated with folate in all women and non-OC-users possibly reflecting erythropoietic activity [43,76].

Confirming previous evidence [36,42,43], our study showed that sTfR was not correlated to the inflammatory biomarker hsCRP. The expected negative correlation of sTfR with ferritin was highly statistically significant [36,43,77].

In our healthy women, ferritin was the only serum iron biomarker unaffected by TAC, OS, inflammation, and OC use; thus, it appears to be the most reliable circulating biomarker to be used in a population of non-pathological fertile age women [32,33,79]. Additionally, the sTfR-F index was unaffected by the abnormal combined TAC/OS condition. It is to be mentioned that determination of TAC and OS status is not part of the routine medical laboratory practice, so that determination of ID is usually performed without knowledge of the oxidative status of the subject. An optimal screening biomarker for ID should also be not affected by common drug treatments like the OC use [80].

### 4.5. Clinical Implications

Our findings highlighted the importance of considering oxidative and inflammatory status in the evaluation of women using oral contraception, because OC use was associated with reduced antioxidant capacity, increased oxidative stress, and increased low-grade inflammation.

An important observation is that in young women hsCRP is strongly associated with low antioxidant defence and high oxidative stress, awareness of this is of interest for clinicians as in clinical routine settings hsCRP is a measure easily available, whereas determination of TAC and/or oxidative stress indicators require specialised laboratories.

Low total antioxidant capacity, elevated oxidative stress and low-grade inflammation are alterations which may represent early biological changes relevant to long-term disease risk, particularly cardiovascular diseases. However, longitudinal studies are required to determine whether these alterations translate into adverse clinical outcomes.

Scientific research indicates that there are complex and important relationships between antioxidant defence, oxidative stress, inflammation, and iron status in the human body [81,82]. Elucidation of the multiple molecular mechanisms connecting these variables require further research [81,82,83]. Evidence suggests that both relevant iron deficiency and iron overload can affect redox state, consequently, proper levels of iron may contribute to oxidative balance [83,84,85].

From a clinical perspective, our findings support the use of ferritin as a reliable ID biomarker among non-pathological women, even in the presence of an altered oxidative condition or OC use.

### 4.6. Limitations and Strengths

This study has several limitations. First, the cross-sectional design does not allow causal inference. Second, the study population consisted of relatively young and healthy women, which may limit generalisability, constraining its applicability to other demographics, including older women or those with comorbidities, and to women with different ethnicities. Third, TAC measurement reflects total antioxidant capacity and does not allow identification of specific antioxidant components. Fourth, future studies should evaluate in detail the modulating effects of lifestyle as dietary habits, variability in antioxidant supplementation, smoking and alcohol intensity, and levels of physical activity. A study performed in young female athletes [15], however, did not observe correlations of OS hydroperoxides with weekly hours of exercise, nor with lifestyle/alimentary habits in OC-users, while in non-OC-users only, OS hydroperoxides were inversely correlated with chocolate and fish consumption. The same study [15] did not find significant differences in FORT and FORD values in supplement users versus non-users among OC-users and among non-OC-users. Fifth, due to the limited number in each type of contraceptive pill, various generations and formulations of oral contraceptives were categorised together; thus, relative variations in oxidative stress and inflammatory reactions were not assessed. However, an investigation examining only third generation contraceptive pills found similar effects of OC use on hsCRP levels [21] of the present study. A study by other authors did not find different levels of lipid peroxide increase according to the type of OC used (mono, bi and tri-phasic pills) [67]. Additionally, an investigation performed in female athletes did not observe differences in FORT and FORD units between OC-users of contraceptive pills containing desogestrel, cyproterone and drospirenone versus pill formulations containing different progestins [15]. Sixth, our study group included only healthy women, none of them had a condition causing an acute phase response, consequently our investigation did not assess the effects of high-grade inflammation on ferritin levels.

The strengths of this study include the well characterised group of participants and the wide number of biomarkers analysed. Inclusion of only healthy women mostly nulliparous avoided the confounding of comorbidities, older age, and pregnancies on oxidative and iron status.

### 4.7. Summary

In summary, our study demonstrated a strong association of OC use with reduced antioxidant capacity, increased oxidative stress, and low-grade inflammation in young women.

In all fertile-age women regardless of OC use, hsCRP was strongly positively associated with oxidative stress, and inversely associated with TAC.

Among iron biomarkers, transferrin and transferrin saturation were modulated by oxidative status and OC use, whereas ferritin, sTfR, and sTfR-F index were not influenced by OC use and TAC/OS imbalance. However, sTfR was affected by hydroperoxides and folate in non-OC-users.

Ferritin emerged as the most independent iron biomarker, unaffected by OC use, oxidative or low-grade inflammatory conditions, and folate values.

## 5. Conclusions

Our study demonstrated that in the healthy young population of fertile women the condition of low TAC is widespread, being particularly present in the vast majority of OC-users, conceivably meaning that these women have low defence against pro-oxidant insults. The pro-oxidant stimuli might derive, for instance, by exposure to UV radiation and pollutants, especially metals as cadmium, lead, mercury [13,26], that in turn may aggravate elevated values of oxidative stress and low antioxidant defence. It is presently not clear whether the condition of low TAC recover after cessation of external hormones’ use, and/or whether some irreversible damages occur by prolonged exposure to low TAC, high OS, and chronic low-grade inflammation. In this respect, some studies on oral contraception and external hormonal treatments observed that women are exposed to increased risk of pathologic conditions associated with oxidative stress like cutaneous melanoma [86], and endometriosis [87] also after cessation of hormonal treatments [88]. Of note, effects on endometriosis have been related to the duration of the hormonal treatment or early age (12–14 years) at OC use [87]. If so, this could mean, for instance, that prolonged exposure to high oxidative stress might determine permanent OS signatures like epigenetic modifications in young people possibly resulting in increased risk of future diseases.

In our opinion, increased awareness might induce young women using OC to minimise exposure to pro-oxidants, and particularly to avoid smoking. Whether the use of antioxidant containing food and/or supplements may attenuate the low TAC, high OS, and the increased low-grade inflammation condition experienced by many OC-users, for instance by enhancing NRF2 a pivotal transcription factor of antioxidant defence [11,59,63], remains to be determined [56].

Regarding blood biomarkers for assessment of iron status in young healthy women, our study observed that ferritin is unaffected by TAC, OS, low-grade inflammation, and folate, whereas sTfR was affected by hydroperoxides in non-OC-users, and by folate in all women and in non-OC-users. Thus, our study showed that among iron status biomarkers, ferritin has the best reliability as a marker of iron status in non-pathological women of reproductive age.

## Data Availability

The original contributions presented in this study are included in the article. Further inquiries can be directed to the corresponding authors.

## Abbreviations

The following abbreviations are used in this manuscript:

BMI	body mass index
CAT	catalase
CI	95% confidence interval
FORD	Free Oxygen Radical Defence
FORT	Free Oxygen Radical Test
GPx	glutathione peroxidases
GSH	reduced glutathione
Hb	haemoglobin
Hct	haematocrit
H_2_O_2_	hydrogen peroxide
HIF-1	hypoxia inducible factor-1
hsCRP	high sensitivity C-reactive protein
ID	iron deficiency
IDA	iron deficiency anaemia
IRPs	iron-regulatory RNA-binding proteins
IREs	iron-responsive elements
IUD	intrauterine device
OC	oral contraception
OH-1	heme oxygenase-1
O_2_·^-^	superoxide anion radical
·OH	hydroxyl radical
NF-ĸB	nuclear factor kappa B
NRF2	nuclear factor erythroid 2-related factor 2
OR	odds ratio
ROOH	organic hydroperoxide
ROS	reactive oxygen species
RNS	reactive nitrogen species
SD	standard deviation
SOD	superoxide dismutase
sTfR	soluble transferrin receptor
sTfR-F index	soluble transferrin receptor (log ferritin)^−1^
Tf	transferrin
TfS%	transferrin saturation percentage
Trolox	6-hydroxy-2,5,7,8-tetramethylchroman-2-carboxylic acid
WBC	white blood cells
